# Tuina plus acupuncture for post-stroke depression: A protocol for systematic review and meta-analysis

**DOI:** 10.1097/MD.0000000000026013

**Published:** 2021-05-21

**Authors:** Meng Meng, Guanyu Hu, Kang Yang, Heran Wang, Yiran Han, Ting Pan, Huijuan Lou, Ye Zhang, Yufeng Wang, Deyu Cong

**Affiliations:** a Department of Acupuncture and Tuina, Changchun University of Chinese Medicine; b Department of Tuina, Traditional Chinese Medicine Hospital of Jilin Province, Changchun, China.

**Keywords:** acupuncture, protocol, post-stroke depression, systematic review, Tuina

## Abstract

**Background:**

Post-stroke depression (PSD) is the most common mental health issue, affecting approximately 33% of stroke survivors. Tuina and acupuncture treatments are often combined to treat PSD; however, there has been no meta-analysis on their synergistic effect. Therefore, we aimed to perform a systematic review and meta-analysis to estimate the effectiveness of Tuina and acupuncture in PSD treatment.

**Methods:**

The following electronic databases will be searched: PubMed, the Cochrane Library, Embase, Web of Science, Medline, CNKI, Chinese Biomedical Literature Database, VIP, and Wan Fang databases. We will consider articles published between database initiation and April 2021. Clinical randomized controlled trials related to Tuina combined with acupuncture for post-stroke depression will be included in the study. Language is limited to Chinese and English. Research selection, data extraction, and research quality assessment were independently completed by 2 researchers. Data were synthesized using a fixed effect model or random effect model, depending on the heterogeneity test. The Hamilton depression rating scale (HDRS) and effective rate were the primary outcomes. The post-stroke depression rating scale (PSDRS), patient health questionnaire-9 (PHQ-9), and the incidence of adverse events will also be assessed as secondary outcomes. RevMan V.5.4 statistical software will be used for meta-analysis. If it is not appropriate for a meta-analysis, a descriptive analysis will be conducted. Data synthesis uses the risk ratio and the standardized or weighted average difference of continuous data to represent the results.

**Results:**

This study provides a high-quality synthesis to assess the effectiveness and safety of Tuina for post-stroke depression.

**Conclusion:**

This systematic review will provide evidence to determine whether Tuina plus acupuncture is an effective and safe intervention for patients with post-stroke depression.

**Ethics and dissemination:**

The protocol of the systematic review does not require ethical approval because it does not involve humans. This article will be published in peer-reviewed journals and presented at relevant conferences.

**Systematic review registration:**

INPLASY202140098

## 1 Introduction

Stroke is the second leading cause of death due to cardiovascular disease.^[1]^ It is also one of the diseases with high mortality and disability rates worldwide.^[2]^ Post-stroke depression (PSD) is the most common mental health issue, affecting approximately 33% of stroke survivors.^[3]^ It is characterized by hopelessness, anxiety, disordered sleep, and reduced responsiveness.^[4]^ PSD has a negative impact on rehabilitation, recuperation of motor and cognitive deficits following stroke, and significantly increases the chances of relapsing neurovascular events.^[5]^ The medical cost of patients with post-stroke depression is about 4 times that of post-stroke patients without depression, which not only severely restricts the ability of patients, reduces the quality of life, but also causes a psychological impact on patients’ rehabilitation, and places a great burden on families and society.^[6]^ An increased risk of suicide and increased mortality has been reported.^[7]^ However, the most clinically important advances have been in the treatment and prevention of PSD. Recent meta-analyses of randomized controlled trials for the treatment of PSD have demonstrated the efficacy of antidepressants.^[8]^ The most effective antidepressants are the selective serotonin reuptake inhibitors escitalopram and paroxetine^[9–12]^; however, they may cause bleeding and intracerebral hemorrhage.^[13]^

Accumulating evidence suggests that brain-derived neurotrophic factor (BDNF) plays a key role in the pathophysiology of depression and PSD.^[14–16]^ The Acupuncture has been practiced in China for >3000 years, and accumulated evidence that acupuncture is beneficial in various conditions significantly enhanced our understanding of the mechanisms of acupuncture treatment.^[17]^ Acupuncture treats nervous system diseases by increasing the levels of brain-derived neurotrophic factor, resulting in neuroprotection, cell proliferation, anti-apoptosis, antioxidant activity, anti-inflammation, and maintenance of the blood–brain barrier.^[18–21]^ Several systematic reviews and meta-analyses have suggested that acupuncture may be superior to antidepressants with respect to clinical effectiveness and alleviation of depressive symptoms in patients with PSD. A review of studies included in these systematic analyses showed that acupuncture combined with Tuina training has been used to treat PSD.

As a complementary and alternative traditional Chinese medicine (TCM) therapy, Chinese Tuina massage, also called Tuina in China, has been widely applied in the clinical treatment of PSD in China for a long time.^[22]^ When moderate and light pressure massage have been compared in laboratory studies, moderate pressure massage reduced depression, anxiety, and heart rate, and altered electroencephalogram patterns, similar to a relaxation response.^[23]^ First, Tuina, through its effect on the skin, can cause local muscle relaxation, increase blood circulation, calm, relieve tension, and treat patients with post-stroke depression.^[24,25]^ Second, existing studies have shown that the comfort generated by local massage can be transmitted to the central nervous system after being felt by the surrounding receptors. Reduced excitability of the central nervous system can improve sleep and relieve anxiety.^[26,27]^ Finally, owing to its reliable efficacy and few side effects, it is widely used in the treatment of various diseases. Many studies have shown that massage can effectively relieve symptoms related to depression.^[28]^ Therefore, the use of acupuncture and Tuina training in PSD therapy requires further research. At present, there is no systematic review of Tuina combined with acupuncture treatment in the treatment of post-stroke depression, so this study will evaluate the efficacy and safety of Tuina combined with acupuncture in the treatment of post-stroke depression, and provide evidence for clinical decision-making of Tuina and acupuncture.

## 2 Methods and analysis

The systematic review will be performed following the guidelines of the Preferred Reporting Items for Systematic Review and Meta-Analysis Protocols (PRISMA-P) 2015.^[29]^ This protocol was registered on the International Platform of Registered Systematic Review and Meta-analysis Protocols (INPLASY202140098).

### 2.1 Inclusion criteria

#### 2.1.1 Types of participants

We will consider patients with a clinical diagnosis of PSD irrespective of their sex, age, severity, and disease duration.

#### 2.1.2 Types of interventions

The treatment group using Tuina while the control group received treatment with oral medication, acupuncture, Chinese herbal medication, physical therapy, botox injections, and so on, or even with no treatment, will be included.

#### 2.1.3 Types of studies

This review will include randomized controlled trials (RCTs) on Tuina for PSD published in Chinese and English. We will exclude non-RCTs, review studies, case reports, and animal experiments.

#### 2.1.4 Types of outcomes

The primary outcomes will include the Hamilton depression rating scale (HDRS) and the effective rate. As previously reported, a ≥25% reduction in the Hamilton depression rating scale score was indicative of effective treatment.

The secondary outcomes were the post-stroke depression rating scale (PSDRS), patient health questionnaire-9 (PHQ-9), and the incidence of adverse events.

### 2.2 Data sources and search methods

#### 2.2.1 Electronic searches

This study will use the PubMed, Cochrane Library, Embase, Web of Science, and Medline databases. In addition, we will also collect 4 databases of China: China National Knowledge Infrastructure, China Biomedical Literature Database, China Science Journal Database, and Wan-fang Database. We will consider articles published between database initiation and April 2021. In addition, we manually retrieve other resources, including the reference lists of identified publications, conference articles, and gray literature. The following search terms will be used: post-stroke depression, PSD, depression, stroke, post-stroke, Tuina, massage, acupuncture, etc. The example search strategy in Table 1 will was used for PubMed. This search strategy is slightly modified and used in several other databases.

**Table 1:** Search strategy for the PubMed database.

Number	Terms
#1	Post-stroke depression (all field)
#2	PSD (all field)
#3	Depression (all field)
#4	#1 OR #2–3
#5	Stroke (all field)
#6	Post-stroke (all field)
#7	Poststroke (all field)
#8	Apoplexy (all field)
#9	Cerebral hemorrhage (all field)
#10	Cerebral infarction (all field)
#11	#5 OR #6–10
#12	Tuina (all field)
#13	Massage (all field)
#14	Acupressure (all field)
#15	Rub (all field)
#16	Massageing (all field)
#17	Massotheraty (all field)
#18	Manipulation (all field)
#19	#12 OR #13–18
#20	Acupuncture
#21	Needle
#22	Auriculo-acupuncture
#23	#20 OR #21–22
#24	Randomised controlled trial (all field)
#25	Controlled clinical trial (all field)
#26	random allocation (all field)
#27	Randomized (all field)
#28	Placebo (all field)
#29	double-blind method (all field)
#30	single-blind method (all field)
#31	#24 OR #25–30
#32	#4 And #11 And #19 And #23 And #31

#### 2.2.2 Searching for other resources

We will search for a list of related references for additional trials. The PubMed and Cochrane Library will be searched for existing systematic reviews related to our topic to search their reference lists for further studies. We will also search a reference list for identifying published journals, books, conference articles, and gray literature related to this research topic.

### 2.3 Data collection and export

**Figure 1:**
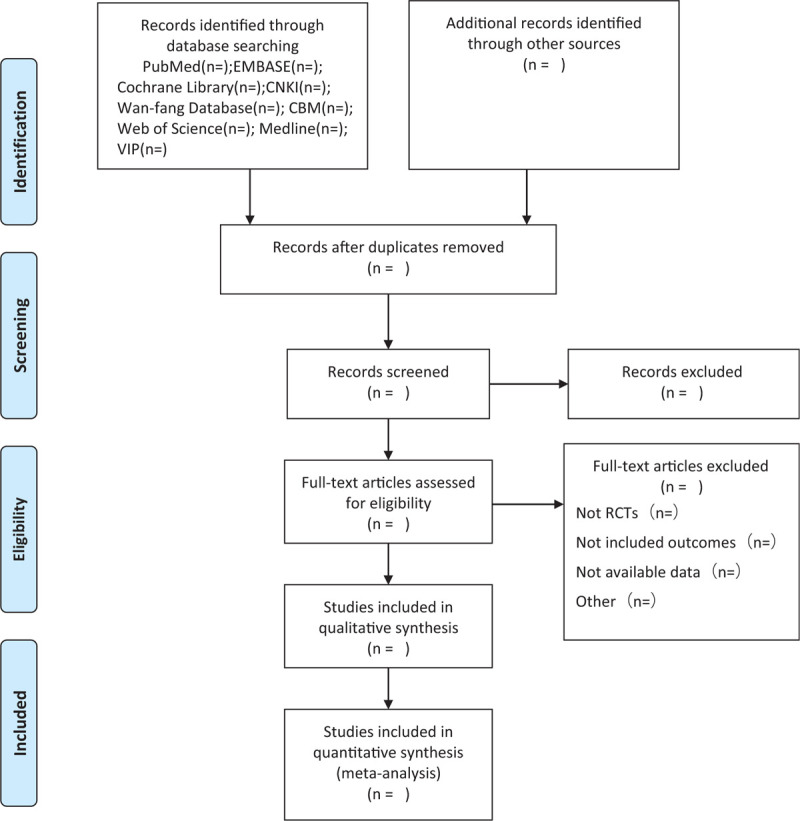
Flow diagram of study selection process.

After completing the search, the results will be exported to Endnote software Version X9, and repetitive studies will be deleted by the software. The process of filtering documents will be completed independently by 2 reviewers and then cross-checked to determine the final inclusion of the documents. In the first stage, all the documents in the search results were screened for titles, abstracts, and keywords to determine which tests met the selection criteria. In the second stage, we will evaluate the full text of the study and determine whether it is eligible for systematic reviews. Studies excluded after reading the full text will also be documented and explained as to why they were excluded. When differences arise at any stage, we invite a third reviewer to discuss arbitration. The research flowchart is presented in Fig. 1.

### 2.4 Data extraction and analysis

Data extraction and analysis will be performed by 2 researchers independently, and the results will be cross-matched. When the differences and opinions are inconsistent, they should be settled through discussion. If the differences encountered cannot be resolved through discussion, a third author will be invited to resolve them. The following information was extracted: journal title, first author, year of publication, study design, patient characteristics, control intervention, experimental intervention, outcomes, duration of intervention, etc. If a study has unclear or inadequate information, we will attempt to contact the authors via email. Any dispute will be resolved by consulting a third reviewer.

### 2.5 Assessment of risk of bias in the included studies

Two reviewers will separately assess the risk of bias of the selected RCTs using the Cochrane risk of bias assessment tool. This tool has the following 7 domains: random sequence generation, allocation concealment, blinding of participants and personnel, blinding of outcome assessment, incomplete outcome data, selective reporting, and other biases. A bias value of “low,” “unclear,” or “high” will be used to rank the risk of bias. These even domains will be separately appraised by 2 reviews, and discrepancies will be addressed by consulting a third reviewer.

### 2.6 Assessment of heterogeneity

The heterogeneity of data was tested by calculating the value of the *I*^2^ statistic. The study was not considered to have a large heterogeneity when the *I*^2^ value was less than 50%. However, when the *I*^2^ value exceeded 50%, there was significant statistical heterogeneity among the trials, and meta-analysis was not performed. At this time, subgroup stratification analysis is needed to explore the possible causes of heterogeneity.

### 2.7 Assessment of reporting biases

We used funnel charts to assess reporting biases. When a sufficient number of included studies (at least 10 trials) are available, we will conduct a test for funnel plot asymmetry using the Egger method.

### 2.8 Data synthesis

Data synthesis will be performed using RevMan V.5.4 (The Cochrane Collaboration, London, United Kingdom). The results are expressed as a risk ratio and the standardized or weighted average difference of continuous data. The specific methods were as follows: if the *I*^2^ test was <50%, the fixed effects model was used for data synthesis. If the *I*^2^ test was between 50% and 75%, the random-effects model was used for data synthesis. If the *I*^2^ test is >75%, we will investigate possible reasons from both clinical and methodological perspectives to conduct a subgroup analysis. If data cannot be synthesized, we provide a descriptive analysis to solve this problem.

### 2.9 Subgroup analysis

In the case of high heterogeneity, we conducted a subgroup analysis to identify the sources of heterogeneity. In addition, according to different course times, or other factors affecting the results, we will also perform a subgroup analysis.

### 2.10 Sensitivity analysis

To test the robustness of the main decisions in the review process, we conducted a sensitivity analysis. The main analysis points included the impact of method quality, sample size, and missing data on the study. The meta-analysis will be reused, and more inferior-quality studies will be excluded. The results were compared and discussed according to the results.

### 2.11 Grading the quality of evidence

The quality of systematic reviews will be evaluated using the grading of recommendations, assessment, development, and evaluation. Five downgrading factors, including risk of bias, inconsistency, indirectness, imprecision, and publication bias were assessed. The assessment results were divided into 4 levels: high, moderate, low, or very low.

## 3 Discussion

Individuals with PSD are at a higher risk of suboptimal recovery, recurrent vascular events, poor quality of life, and mortality.^[30]^ At present, antidepressants are considered to be effective drugs for the treatment of PSD, but due to the serious side effects and high price of drug treatment, patients do not adhere to the treatment, leading to the aggravation of post-stroke depression symptoms. Tuina and acupuncture therapy, as a commonly used adjuvant therapy, can be a good treatment for stroke patients with depression symptoms, which can also play a role in comprehensive conditioning, sleep, anxiety, emotional tension, and other problems can be well treated. In addition, this combination therapy can enhance the efficacy, shorten the treatment period, and are simple to operate with fewer side effects, making it the preferred treatment option for PSD. This systematic review will focus on the efficacy and safety of Tuina combined with acupuncture for post-stroke depression. Tuina and acupuncture are traditional Chinese physical therapies that are effective for a variety of diseases in China, including depression.^[31]^ Clinical reports show that Tuina and acupuncture are effective in the treatment of post-stroke depression; however, high-quality studies have not yet been conducted. We conducted this review to provide better evidence and guidance for clinical decision-making.

## Author contributions

**Data curation:** Heran Wang, Yiran Han.

**Formal analysis:** Huijuan Lou, Ye Zhang.

**Funding acquisition:** Deyu Cong.

**Investigation:** Kang Yang, Ting Pan.

**Methodology:** Guanyu Hu.

**Validation:** Yufeng Wang.

**Writing – original draft:** Meng Meng.

**Writing – review & editing:** Yufeng Wang, Deyu Cong.
